# Supplementary material for: Symbionts shape host innate immunity in honeybees
Source: Proc Biol Sci. 2020 Aug 26;287(1933):20201184. doi: 10.1098/rspb.2020.1184 (PMC7482289; doi:10.1098/rspb.2020.1184)
Supplement: Supplementary Figures and Table [file rspb20201184supp1.pdf]

Supplementary figures for the article:

Title: Symbionts shape host innate immunity in honey bees

Authors: Richard D. Horak[1*], Sean P. Leonard[1], Nancy A. Moran[1*]

Affiliations: [1]Department of Integrative Biology, The University of Texas at Austin, Austin, Texas 78712, United States

***Correspondence to:** rdhorak1@gmail.com, nancy.moran@austin.utexas.edu

DOI: 10.1098/rspb.2020.1184

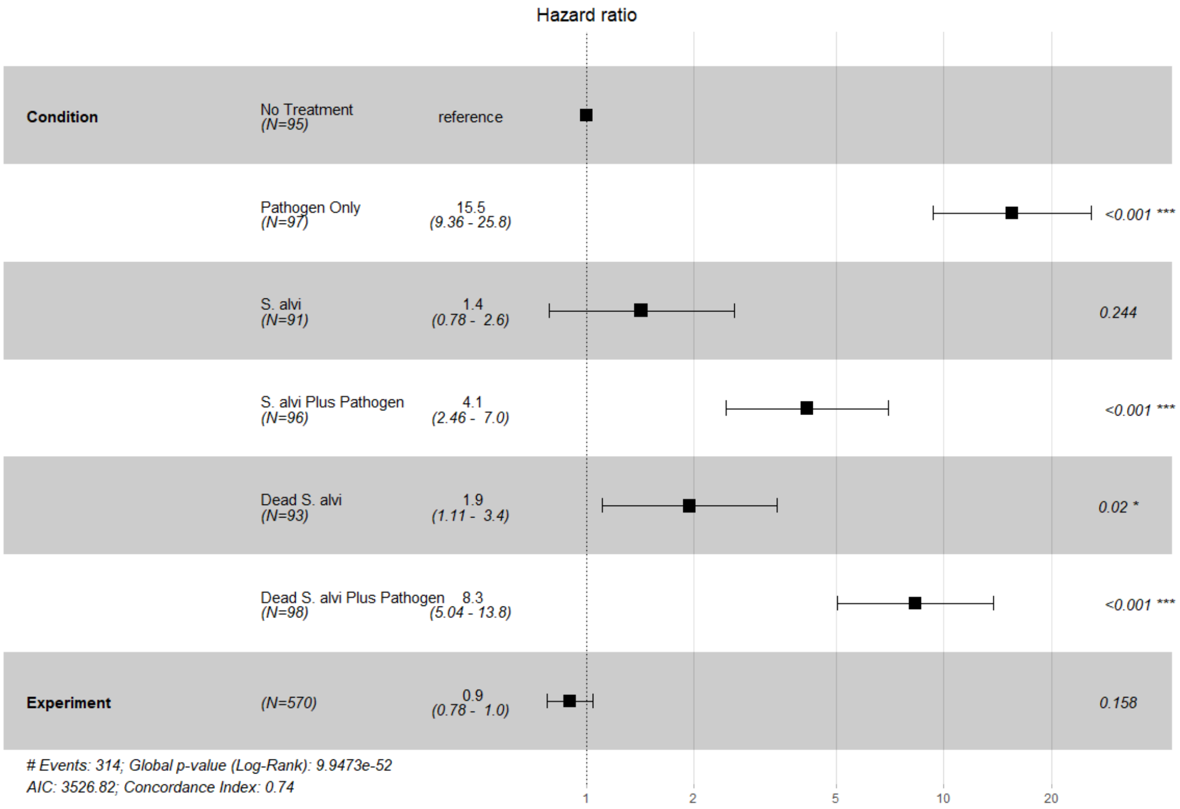

Supplementary Figure 1 Bees challenged by the pathogen *S. marcescens* have higher survival when pre-inoculated with *S. alvi*

Hazard plot detailing effect of honey bee treatment and replicate experiments on honey bee survival after inoculation with PBS or 5 ul of the pathogen *S. marcescens* at OD_{600} = 1. Honey bees given no bacterial inoculum and no pathogen exposure used as a reference group. Bees treated with live *S. alvi* or heat-killed *S. alvi* without pathogen exposure were affected much less than honey bees exposed to the pathogen, regardless of prior treatment. Total N = 570 bees.

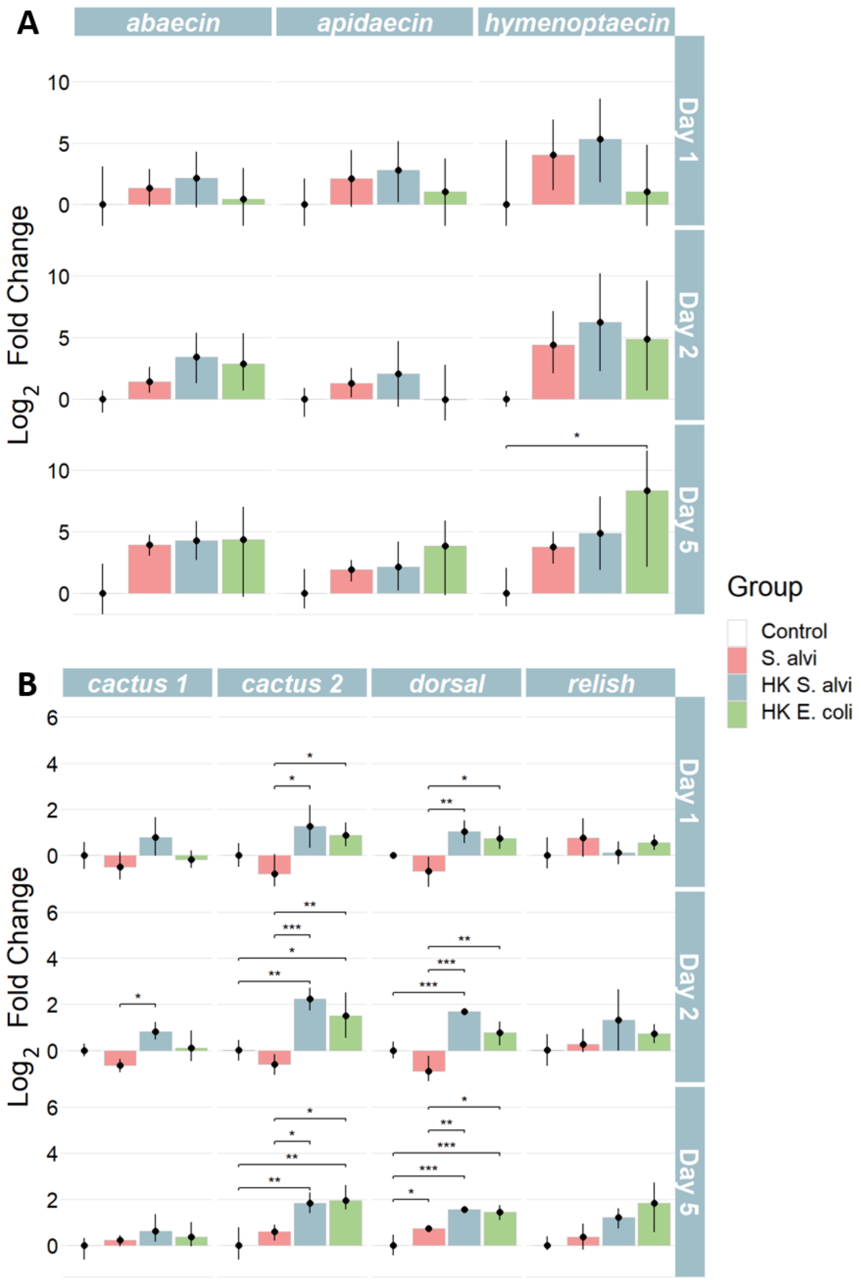

A
abaecin
apidaecin
hymenoptaecin
Day 1
Day 2
Day 5
Log2 Fold Change
10
5
0
B
cactus 1
cactus 2
dorsal
relish
6
4
2
0
Group
Control
S. alvi
HK S. alvi
HK E. coli

Supplementary Figure 2 Live and heat-killed *S. alvi* trigger differential host immune gene expression

Bee gene expression relative to the housekeeping gene *RPS18* measured using qPCR from cDNA derived from bee whole abdomens 1,2, and 5 day(s) post treatment. HK = heat-killed. (A) Bees inoculated with live *S. alvi*, heat-killed *S. alvi*, and heat-killed *E. coli* trigger non-significant upregulation of AMPs compared to the uninoculated control group. (B) Bees inoculated with heat-killed *S. alvi* or *E. coli* had significantly higher expression of *cactus2* and *dorsal* than the control or live *S. alvi* groups. Total N = 54 bees from one hive. *** = P < 0.001, ** = P < 0.01, * = P < 0.05. P-values obtained using Tukey Honest Significant Difference method.

Supplementary Table 1 qPCR primer sequences for gene expression analysis

Gene	Source	Forward	Reverse
**Abaecin**	[23]	TCGGATTGAATGGTCCCTGAC	ATCTTCGCACTACTCGCCAC
**Apidaecin**	[23]	GTAGGTCGAGTAGGCGGATCT	TTTTGCCTTAGCAATTCTTGTTG
**Cactus-1**	[53]	CTATCGTGGAGAAACTGCGTAT	TCAGGAAGTGGTTCTGGTATTG
**Cactus-2**	[53]	ATCAGACGGCTCTGCTCTAT	TCGTCTTCGTCAGTGGTATCT
**Dorsal**	[54]	AGAGATGGAACGCAGGAAAC	TGACAGGATATAGGACGAGGTAA
**Dredd**	[54]	GCGTCATAAAGAAAAAGGATCA	TTTCGGGTAATTGAGCAACG
**Hymenoptaecin**	[55]	GTCGTCCATCCTTGGACATT	TTTCCCAAACTCGAATCCTG
**PGRP-LC**	[54]	TCCGTCAGCCGTAGTTTTTC	CGTTTGTGCAAATCGAACAT
**Pirk**	This Study	GCCCGAAATCTAGCAAGGATAA	TTCCTCTCCTCGTCCATCTT
**Relish**	[55]	GGAGCTGATCCAAATCGAAC	AGTGGCATCCATCCATCATT
**RPS18**	[53]	AGGTGTTGGTCGTCGTTAT	CATTCTCCAGCACGCTTAT
**Toll**	[54]	TAGAGTGGCGCATTGTCAAG	ATCGCAATTTGTCCCAAAAC
